# Homeostatic regulation of neuronal function: importance of degeneracy and pleiotropy

**DOI:** 10.3389/fncel.2023.1184563

**Published:** 2023-06-02

**Authors:** Jane Yang, Steven A. Prescott

**Affiliations:** ^1^Neurosciences and Mental Health, The Hospital for Sick Children, Toronto, ON, Canada; ^2^Institute of Biomedical Engineering, University of Toronto, Toronto, ON, Canada; ^3^Department of Physiology, University of Toronto, Toronto, ON, Canada

**Keywords:** degeneracy, pleiotropy, excitability, ion channels, homeostatic regulation, robustness

## Abstract

Neurons maintain their average firing rate and other properties within narrow bounds despite changing conditions. This homeostatic regulation is achieved using negative feedback to adjust ion channel expression levels. To understand how homeostatic regulation of excitability normally works and how it goes awry, one must consider the various ion channels involved as well as the other regulated properties impacted by adjusting those channels when regulating excitability. This raises issues of degeneracy and pleiotropy. Degeneracy refers to disparate solutions conveying equivalent function (e.g., different channel combinations yielding equivalent excitability). This many-to-one mapping contrasts the one-to-many mapping described by pleiotropy (e.g., one channel affecting multiple properties). Degeneracy facilitates homeostatic regulation by enabling a disturbance to be offset by compensatory changes in any one of several different channels or combinations thereof. Pleiotropy complicates homeostatic regulation because compensatory changes intended to regulate one property may inadvertently disrupt other properties. Co-regulating multiple properties by adjusting pleiotropic channels requires greater degeneracy than regulating one property in isolation and, by extension, can fail for additional reasons such as solutions for each property being incompatible with one another. Problems also arise if a perturbation is too strong and/or negative feedback is too weak, or because the set point is disturbed. Delineating feedback loops and their interactions provides valuable insight into how homeostatic regulation might fail. Insofar as different failure modes require distinct interventions to restore homeostasis, deeper understanding of homeostatic regulation and its pathological disruption may reveal more effective treatments for chronic neurological disorders like neuropathic pain and epilepsy.

## Introduction

**FIGURE 1:**
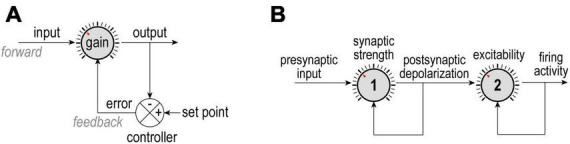
Negative feedback control. **(A)** The difference between an output and its set point constitutes an error signal that is used to adjust how the input is processed. Homeostatic adjustments maintain output near its set point despite changes in input; for example, ion channel levels are adjusted to maintain a desired average firing rate despite changes in presynaptic activity. **(B)** In a more complicated scenario, presynaptic activity (input) is modulated by synaptic strength (gain 1) to yield post-synaptic depolarization (output 1 = input 2), which is, in turn, modulated by intrinsic excitability (gain 2) to yield firing rate (output 2). Here, each dial is controlled independently using separate error signals, but other scenarios are conceivable. Understanding how each loop is organized and whether loops interact is important for appreciating what is being regulated and how.

Homeostasis refers to a property being maintained at or near a set point despite changing conditions. For example, the human body strives to maintain its internal temperature near 37°C despite fluctuations in air temperature. Similar regulation occurs at different biological scales and for diverse properties. First articulated in modern form by Claude Bernard, the concept of homeostasis was formalized by Walter Cannon (who also coined the term) and has benefited from advances in control theory in applied mathematics and engineering (O’Leary and Wyllie, 2011; Billman, 2020). Output of a homeostatically regulated system is compared against a target value, or set point, to calculate an error signal which is used to adjust system parameters so that output is kept near the set point (Figure 1A). For example, in thermoregulation, changes in air temperature trigger shivering or sweating to increase or decrease body temperature. Likewise, the furnace or air conditioning automatically turn on and off to regulate room temperature. Temperature reflects the cumulative output of the furnace or air conditioner minus ongoing heat exchange through poorly insulated windows, etc., making the feedback integral in nature. The same is true for neuronal excitability, which depends on the cumulative insertion of sodium, potassium, and other channels into the cell membrane minus ongoing turnover of those channels. Here, compensatory changes in those channels serve to maintain firing rate near its set point despite disruptive changes in the synaptic input or expression/function of other ion channels.

Throughout this paper we refer to *regulation* of system output via *control* or adjustment of system parameters; in other words, firing rate (output) is regulated to a set point by adjusting synaptic weights or ion channel densities (parameters). Stability (of output) through change (of parameters) is the definition of allostasis, but this is an implicit feature of homeostasis rather than a distinct concept (see also Day, 2005; O’Leary and Wyllie, 2011). The distinction between regulation and control can nevertheless become murky; for instance, one could view excitability as a property to be regulated (by adjusting ion channel densities) or as a parameter to be controlled (to offset changes in synaptic input in order to maintain firing rate). We treat excitability as a regulated property because of the context in which we consider it.

Unlike the simple block diagram shown in Figure 1A, complex biological systems involve multiple feedback loops regulating diverse properties and operating on many spatial and temporal scales. The coexistence of multiple feedback loops means they might interact. Feedback loops can be nested in a hierarchical fashion or arranged in series, with the output of one serving as input to the next. For example, if presynaptic activity (input) is reduced or increased, its post-synaptic effect can be amplified or attenuated by dialing up or down synaptic strength (gain) to produce comparable depolarization (output); in turn, depolarization (input) can be amplified or attenuated by dialing up or down intrinsic excitability (gain) to help maintain firing rate (output) (Figure 1B). In other words, synaptic strength and excitability are adjusted by separate dials arranged in series (Turrigiano, 2011). This is important insofar as treating presynaptic activity as input and firing rate as output but considering only one of the two intervening dials will yield an incomplete and potentially confusing picture. Moreover, the feedback loops controlling those dials might share certain elements, introducing crosstalk that can further obfuscate operations.

**FIGURE 2:**
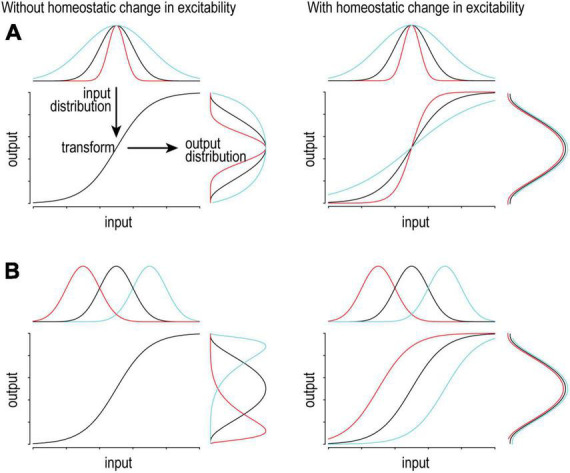
Adjusting excitability supports consistent coding. On a short timescale, variations in input will produce (and be encoded by) variations in output firing. Ideally, the input distribution matches the dynamic range of the system so that the full range of output is used to represent the full range of input. To maintain optimal coding, the input-output transformation (excitability) should adapt to slow changes in the input distribution in order to maintain the output distribution. **(A)** If the input distribution is compressed (red) or expanded (cyan), gain should increase or decrease, respectively. **(B)** If the input distribution is shifted left (red) or right (cyan), offset should be shifted left or right, respectively. Notably, different changes in the input call for different homeostatic changes in the transformation.

One must appreciate that transient changes in synaptic input drive neurons to increase or decrease their firing rate. In that regard, firing rate modulation contributes to neural coding and it would be counterproductive for homeostatic regulation to blunt that modulation. However, a sustained change in firing rate due to a sustained change in input or for some other reason (e.g., chronic sodium channel blockade or elevated extracellular potassium) will trigger homeostatic changes. Slow homeostatic changes support neural coding by adjusting dynamic range so that transient changes in firing rate can effectively encode transient changes in input, lest coding be compromised by a ceiling or floor effect (Figure 2). Different changes in the input distribution benefit from distinct compensatory changes in excitability to optimize coding (compare Figures 2A, B), highlighting the potential benefits of different types of excitability regulation, though experiments have yet to resolve whether those different types occur. That said, neurons must also balance their energy budget, control their osmolarity and volume, and presumably maintain many other properties within acceptable bounds, thus highlighting the need for many feedback loops to operate together (Frere and Slutsky, 2018; Styr et al., 2019; Yang et al., 2022).

In this review, we highlight several concepts that are crucial for understanding the context in which negative feedback operates. We then demonstrate how those concepts apply, especially when multiple properties are co-regulated. We finish by discussing various ways in which homeostasis can go awry and the implications for strategic intervention.

## Homeostatic regulation is widespread but diverse

As illustrated in Figure 1B, homeostatic processes can regulate intrinsic excitability (Desai et al., 1999) and synaptic strength (O’Brien et al., 1998; Turrigiano et al., 1998) as well as the thresholds for inducing synaptic plasticity (Kirkwood et al., 1996) (for reviews, see Davis and Bezprozvanny, 2001; Turrigiano, 2008, 2011, 2012; Davis, 2013; Gainey and Feldman, 2017; Keck et al., 2017; Lee and Kirkwood, 2019). Homeostatic regulation of synaptic strength, or synaptic scaling, has garnered the most attention. It has been demonstrated in pyramidal cells in culture (Turrigiano et al., 1998) and *in vivo* (Desai et al., 2002), and has been observed in layers 2/3, 4, and 5 of several cortical areas, although differences exist, for instance, in developmental regulation (see below). Even within the same layer, different types of pyramidal neurons exhibit differences in their synaptic scaling (Greenhill et al., 2015; Pandey et al., 2022). Homeostatic regulation has also been described in inhibitory synapses and cells (Kilman et al., 2002; Shao et al., 2013; Barnes et al., 2017), including basket cells (Gainey et al., 2018) and chandelier cells (Pan-Vazquez et al., 2020). The balance of synaptic excitation and inhibition is critical for network function, and relies on appropriate regulation of excitatory and inhibitory synapses and neurons. Indeed, homeostatic regulation of intrinsic excitability occurs in both pyramidal neurons (Desai et al., 1999) and inhibitory interneurons (Gainey et al., 2018). Regulation of the axon initial segment, which plays a key role in action potential initiation, is notable (Grubb and Burrone, 2010; Kuba et al., 2010; Wefelmeyer et al., 2016).

The aforementioned work was conducted in rodents but homeostatic regulation has also been demonstrated in invertebrates, including lobster (Turrigiano et al., 1994) and *Drosophila* (Davis and Goodman, 1998; Baines et al., 2001), and in other vertebrate species, including human neurons (Zhang et al., 2018). Most of the vertebrate studies were conducted in cortical neurons but homeostatic regulation has also been described in the retina (Tien et al., 2017) and spinal cord (O’Brien et al., 1998). Even the neuromuscular junction exhibits homeostatic changes (Galante et al., 2000). This overview is not comprehensive but suffices to demonstrate that homeostatic regulation is widespread. Homeostatic mechanisms are not necessarily equivalent across different cells or synapses, or even within the same cell or synapse over the course of development, but general principles (see Figure 1) tend to be shared.

Homeostatic regulation is critical for development (Turrigiano and Nelson, 2004; Tien and Kerschensteiner, 2018), during which major changes occur such as the formation of new synapses and shifts in chloride reversal potential that profoundly alter synaptic inhibition. These and other developmental changes necessitate homeostatic adjustments to maintain and optimize circuit function. Homeostatic regulation occurs in adulthood, but not necessarily the same as during development. For example, synaptic scaling in layer 4 of visual cortex is limited to the critical period (Desai et al., 2002) whereas synaptic scaling in layer 2/3 continues into adulthood (Goel and Lee, 2007). Whereas synaptic scaling in layer 2/3 persists into adulthood, homeostatic regulation of the intrinsic excitability of layer 2/3 neurons is limited to the critical period (Wen and Turrigiano, 2021). Furthermore, suppressing activity triggers homeostatic changes only if applied after synapse formation (Burrone et al., 2002) and the direction of homeostatic regulation switches in accordance with the switch in polarity of chloride flux at axo-axonic synapses (Pan-Vazquez et al., 2020). For the remainder of this article, we will gloss over these mechanistic details, focusing instead on general principles.

## Degeneracy, pleiotropy, and other key concepts

**FIGURE 3:**
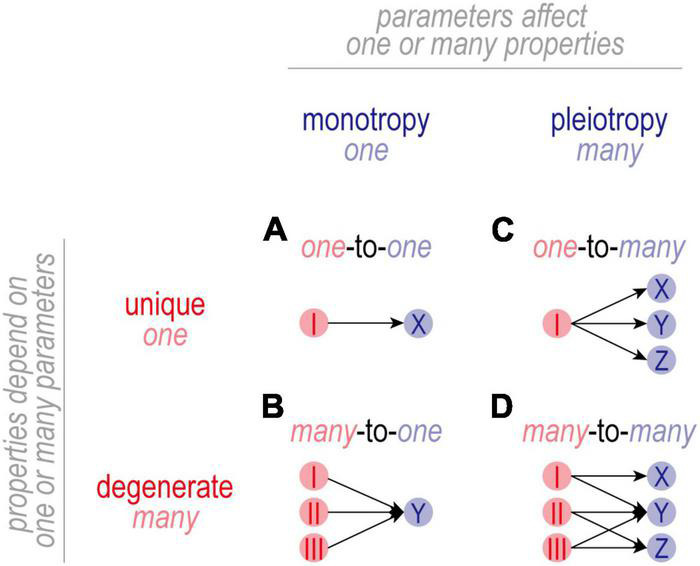
Mapping between parameters and properties. **(A)** In one-to-one mapping, one parameter affects one property. The basis (or solution) for property *X* is unique in that it depends only on parameter *I*. Parameter *I* is monotropic in that it affects only property *X*. **(B)** In many-to-one mapping, the basis for property *Y* is degenerate in that the same value of *Y* can be achieved with multiple different combinations of parameters *I-III*. Individual parameters are still monotropic in that they affect only property *Y*. **(C)** In one-to-many mapping, one parameter affects multiple properties. While the basis for properties *X-Z* is unique, parameter *I* is pleiotropic in that it affects properties *X-Z*. In this scenario, adjusting parameter *I* to regulate property *X* risks disrupting properties *Y* and *Z*. **(D)** Many-to-many mapping combines degeneracy and pleiotropy. Despite the disruptive consequences of adjusting a pleiotropic channel, there is usually a channel combination that will yield the intended value for all properties because a degenerate property can achieve its intended value using different channel combinations. In this scenario, adjusting one channel is liable to trigger secondary adjustments in many other channels. Degeneracy makes it possible for combined changes to settle on mutually agreeable solutions, thus enabling multiple properties to be co-regulated by adjusting pleiotropic channels.

**FIGURE 4:**
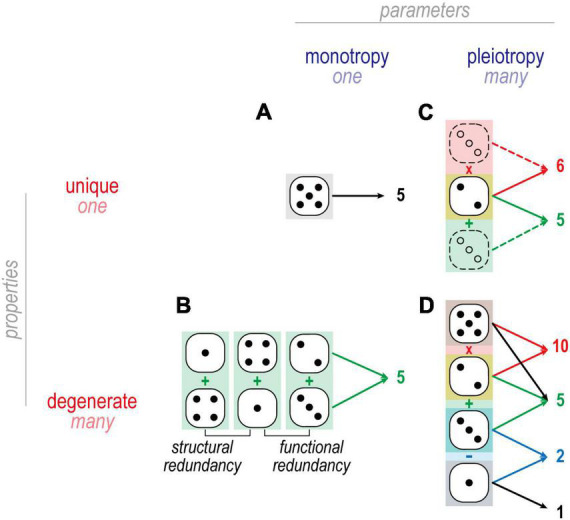
Dice analogies to illustrate mappings in Figure 3. **(A)** An example of one-to-one mapping is when one die is rolled to produce 5. There is only one way to throw a 5 when using a single die. **(B)** An example of many-to-one mapping is when two dice are summed to produce 5: 4 + 1 and 2 + 3 are functionally but not structurally redundant and thus constitute degenerate solutions. By comparison, 4 + 1 and 1 + 4 are structurally redundant, and do not constitute degenerate solutions. **(C)** An example of one-to-many mapping is when one die affects two different arithmetic operations, such as 2 on one die combining with 3 on another to yield 5 (=2 + 3) and 6 (=2 × 3). **(D)** In many-to-many mapping, multiple dice combine in different ways to produce multiple outcomes. One may recognize this as a system of linear equations, e.g., which combination of dice adds to give *X* and multiplies to give *Y*. Each unknown constitutes a degree of freedom and each equation constitutes a constraint. If constraints outnumber degrees of freedom, no solution likely exists and the system is said to be overdetermined. If degrees of freedom outnumber constraints, many solutions likely exist and the systems is therefore undetermined. From the perspective of a homeostatically regulated system, underdetermination is beneficial since any solution giving the desired output is acceptable, and so having many acceptable solutions makes for easier, more robust regulation.

To more fully understand the negative feedback depicted generically in Figure 1A, one must consider which parameters (ion channel densities) are adjusted and what properties in addition to excitability are impacted by those adjustments. As summarized below and illustrated in Figures 3, 4, parameters can map to properties in different ways, with important implications.

In one-to-one mapping (Figure 3A), one parameter affects one property. The basis (or solution) for property *X* is unique in that it depends solely on the value of parameter *I*. Parameter *I* is monotropic in that it affects only property *X*. The resulting regulation is straightforward to understand but is not very robust insofar as regulation of *X* is entirely reliant on properly adjusting *I*; if *I* reaches an upper or lower bound (i.e., saturates), or if *I* itself is compromised, there is no recourse and regulation of *X* will fail.

In many-to-one mapping (Figure 3B), the basis for Property *X* is no longer unique and is instead said to be degenerate. Under these conditions, property *X* can achieve its target value using different combinations of values of parameters *I-III*. This increases robustness insofar as compensatory changes can be distributed across multiple parameters, reducing the reliance on properly adjusting any one parameter. It should go without saying, but a causal relationship between parameter *I* and property *X* does not exclude parameters *II* and *III* from also affecting *X*. By extension, claiming that changes in *I* are necessary and sufficient for changes in *X* hinges on *II* and *III* not changing. If unmeasured, the status of parameters *II* and *III* constitute known unknowns. One must also be wary of unknown unknowns: what if property *X* also depends on other, unidentified parameters?

In one-to-many mapping (Figure 3C), a parameter affects more than one property and is said to be pleiotropic. For the example shown, the bases for properties *X-Z* are unique but parameter *I* is pleiotropic in that it affects all three properties. Pleiotropy complicates co-regulation of multiple properties because adjusting *I* to regulate *X* risks disrupting *Y* and *Z* (and potentially other unidentified properties). Such complications go unrecognized if one focuses on regulation of *X* without considering the consequences for *Y* and *Z*. If all three properties are (co-)regulated, then regulation of property *X* is more constrained than superficial analysis might suggest.

Many-to-many mapping (Figure 3D) capitalizes on the benefits of degeneracy and solves the complications introduced by pleiotropy, but the resulting regulation is anything but straightforward. For the example shown, *X* depends uniquely on *I* whereas *Y* and *Z* depend (degenerately) on different combinations of *I-III*. This degeneracy means that certain combinations of parameters *I-III* are *functionally* redundant, but parameters *I-III* themselves are not functionally redundant in that they each affect different properties. Instead, parameters *I-III* are said to functionally overlap on property *Y*, and parameters *I* and *II* functionally overlap on property *Z*; there is no functional overlap on property *X*. Functional overlap requires that properties are degenerate and that parameters are pleiotropic, which is synonymous with many-to-many mapping.

Functional overlap can also be considered from a different, more intuitive perspective. Channels with similar gating characteristics (voltage-dependencies, kinetics, etc.,) are liable to impact the same cellular properties (Goaillard and Marder, 2021). In that regard, functional overlap naturally derives from similarities in ion channel gating. But this can be deceiving. For instance, the sodium channels Na_*V*_1.7 and Na_*V*_1.8 both activate during action potentials, but because it activates at voltages near threshold, Na_*V*_1.7 is typically ascribed an important role in spike initiation whereas Na_*V*_1.8, because it activates at suprathreshold voltages, is thought to contribute exclusively to the depolarizing upswing of the spike, only after initiation has occured (Bennett et al., 2019); in that sense, the two channels are functionally distinct. However, in the absence (or upon inactivation) of Na_*V*_1.7, voltage threshold shifts into the range where Na_*V*_1.8 activates, allowing Na_*V*_1.8 to contribute to spike initiation (Xie et al., 2022), thus revealing greater functional overlap than comparison of their voltage-sensitivities suggests and, more generally, that functional overlap can be context-dependent.

The term redundancy is often used interchangeably with degeneracy. True redundancy refers to solutions that are functionally and structurally equivalent (Figure 4); for example, a building may receive two power lines so that electricity is not lost if one of the two lines is compromised. Degeneracy refers to solutions that are functionally equivalent but structurally distinct; for example, a building may have one power line and one generator to protect against loss of electricity. Because structurally distinct solutions have different susceptibilities (i.e., are likely to fail for different reasons), degeneracy typically conveys greater robustness than true redundancy; for example, both power lines might be compromised during a hurricane, in which case a generator conveys more robustness than a second power line. In the case of neuronal excitability, degeneracy means that ion channels can combine in different ways to yield equivalent excitability; for example, the same output might be achieved with channels *I*, *II*, and *III* expressed with ratio 20:20:60 or 0:20:80 or 50:50:0. Consequently, a given channel type might be expressed at very different levels in different (yet equally excitable) cells so long as all the channel types in each cell are “balanced.” That balancing leads to ion channel correlations (see the section “Homeostatic control of ion channels affecting excitability”).

Building on the concept of robustness, degeneracy is a prerequisite for evolution insofar as it facilitates acquisition of new functionality by preventing disruption of existing functionality (Edelman and Gally, 2001). If a gene serving a certain function is duplicated, that function can rely on two independent (but initially identical) genes. One of those genes is then free to mutate without compromising the original function, and, in so doing, may achieve new functionality. For example, all cells must control the flux of ions across their membrane to regulate their volume, which depends on osmotic forces; but as channels, pumps and co-transporters increased in number and diversity, neurons could exploit ion flux for signaling without compromising their volume. This also explains pleiotropy: ion channels evolve to affect new properties without (completely) losing their effect on “initial” properties. By this logic, duplicating genes (to produce redundancy) is necessary but not sufficient to expand functionality; instead, random variations are also necessary, but by having their disruptive effects mitigated by redundancy, random variations are more likely to have a net beneficial effect and be selected for. This results in degenerate solutions. Pleiotropy and functional overlap are natural byproducts of this scheme.

Homeostatic regulation of multiple properties using pleiotropic components is effective but not straightforward to understand. For instance, adjusting a certain ion channel to regulate one property risks disrupting other properties, just as adjustments made to regulate those other properties may disrupt the first property. The crosstalk is bidirectional if the same ion channel is involved in regulating two (or more) properties, meaning different negative feedback loops that converge on the same ion channel may try to adjust the expression of that channel in opposite directions, or at least to different degrees. The crosstalk is unidirectional if an ion channel affects two properties but is adjusted by only one of the feedback loops. In any case, other channels controlled by the feedback loops of affected properties will need to undergo compensatory changes that simultaneously restore multiple properties to their respective set point (Olypher and Calabrese, 2007; Yang et al., 2022). This difficult task, which requires degeneracy, introduces correlations in channel expression and has notable consequences for the organization of negative feedback loops.

## Homeostatic control of ion channels affecting excitability

Neurons adjust their ion channels in order to maintain a stable firing rate and specific activity patterns like rhythmic bursting (Turrigiano et al., 1994; Desai et al., 1999; Golowasch et al., 1999; Baines et al., 2001; Brickley et al., 2001; Mee et al., 2004; Swensen and Bean, 2005; O’Leary et al., 2010; Amendola et al., 2012). The consistency of function is seemingly inconsistent with the variability in expression of a given channel across neurons (Schneider et al., 2022). That paradox is explained by the co-variation of other channels in the same neuron (Schulz et al., 2007; Tobin et al., 2009; Zhao and Golowasch, 2012; Gaiteri et al., 2014; Temporal et al., 2014; Tapia et al., 2018; Santin and Schulz, 2019; Tran et al., 2019; Kodama et al., 2020). Correlations arise from activity-dependent control, as demonstrated in stomatogastric ganglion neurons by Santin and Schulz (2019), who showed, after removing synaptic and modulatory inputs, that the majority of channel mRNA correlations were restored by artificially re-introducing activity patterns. In simulations, O’Leary et al. (2013, 2014) showed that correlations reflect the relative rates with which different channels are adjusted (see also Mishra and Narayanan, 2021).

Notably, only positive correlations in mRNA levels have been reported (Schulz et al., 2007; Tobin et al., 2009; Temporal et al., 2014; Tapia et al., 2018; Santin and Schulz, 2019; Kodama et al., 2020) although experiments and simulations predict that negative correlations should also occur or, more specifically, that negatively correlated conductance densities can produce target outputs (Hudson and Prinz, 2010; Soofi et al., 2012; Zhao and Golowasch, 2012; O’Leary et al., 2013; Jain and Narayanan, 2020; Yang et al., 2022). Interestingly, naturally occurring negative correlations were reported by Khorkova and Golowasch (2007), but they measured conductance densities rather than mRNA levels. This hints that post-transcriptional processes (translation, membrane trafficking, etc.,) may also introduce correlations. Negative correlations in mRNA levels have been reported in *Drosophila* but genes with negatively correlated expression are less likely to share a transcription factor binding site than those with positively correlated expression (Marco et al., 2009). Evidence suggests that translational control may also help mediate homeostatic regulation (Baines, 2005), consistent with the effects of translational repressors like pumilio (Mee et al., 2004) and the fragile X messenger ribonucleoprotein, FMRP (Richter et al., 2015). Having a multiplicity of dials is beneficial if not necessary to co-regulate multiple properties (see below), and so it might be expected that ion channel expression is controlled at multiple levels. Correlations in conductance densities are ultimately what is important for neuronal excitability, but correlations at intermediate levels can nevertheless help disentangle the negative feedback loops (Gaiteri et al., 2014).

**FIGURE 5:**
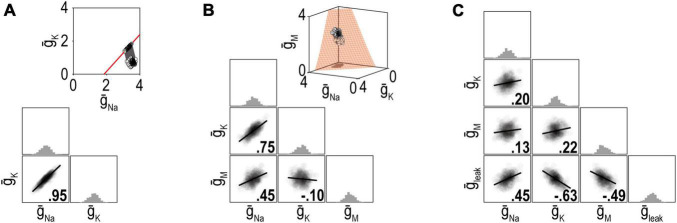
Dimensionality of the solution manifold affects the strength of pairwise correlations. For insets in panels **(A,B)**, all parameter combinations producing the desired firing rate of 40 spk/s are shown in red and constitute the solution manifold. The solution manifold corresponds to a curve in panel **(A)** (1-dimensional) and a surface in panel **(B)** (2-dimensional). Dots show a set of ion channel combinations initially (white) and after regulation (gray). Other plots summarize distributions of channel densities after regulation. **(A)** When firing rate is regulated by adjusting just two ion channels, the pairwise correlation is strong because variation in one channel is offset entirely by co-variation in the other channel. **(B)** When firing rate is regulated by adjusting three channels, pairwise correlations weaken because variation in one channel is offset by variations in two other channels. **(C)** Pairwise correlations continue to weaken as more adjustable channels are involved. Ion channel correlations may exist despite a high-dimensional solution manifold if the homeostatic regulation maintain correlations despite noise (see text). Modified from Figure 6 of Yang et al. (2022).

Ion channel correlations reflect different density combinations yielding the same output (i.e., degenerate solutions). The degree of degeneracy, which is reflected in the dimensionality of the solution manifold, affects the strength of correlations (Figure 5). Specifically, pairwise correlations are necessarily strong if the solution manifold is low-dimensional (because disruptions are offset by compensatory changes in one or a few channels) but they can be weaker if the solution manifold is high-dimensional (because disruptions can be offset by compensatory changes distributed across many channels). Whether solutions distribute across the solution manifold or remain within a restricted location, and retain their correlations, depends on details of the regulation mechanism. O’Leary et al. (2013) explained how correlations reflect the angle at which solutions approach the solution manifold, but Franci et al. (2020) subsequently highlighted how noise spreads those solutions across the manifold unless cooperative interactions prevent this. Spreading occurs because the error signal is zero everywhere on the solution manifold; therefore, negative feedback brings solutions to the manifold but cannot limit their spread across the manifold.

Correlations in the densities of different channels explain why the density of any one channel can vary so much without disrupting neuronal function—because activity-dependent control introduces co-variations in other channels. This highlights an important point: the expression level of a given channel considered in isolation says little about the excitability of a neuron, and vice versa, neuron excitability is not explained by the expression level of any one channel. The broader context, namely, expression levels of all the channels affecting excitability, must be factored in. Furthermore, because channel expression is correlated within each neuron, one cannot measure different channels in different neurons and cobble those measurements together to infer a generic neuron. This failure of averaging has been pointed out before (Golowasch et al., 2002) but remains underappreciated. For such averaging to work, the densities of different channels must be independent, which is precisely what activity-dependent control prevents.

## Multiple properties are regulated concurrently

**FIGURE 6:**
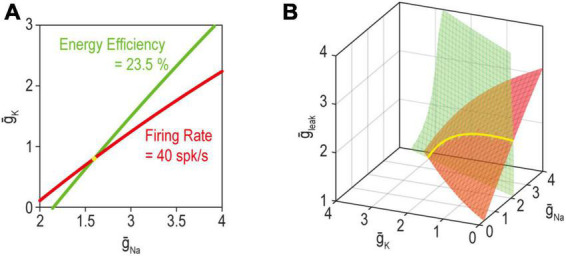
The solution for multiple properties corresponds to where the individual solutions for each property intersect. **(A)** When adjusting just two ion channels, the solution manifold for firing rate (red) or energy efficiency (green) each correspond to a curve (1-dimensional). Hence, the joint solution for both properties (yellow) corresponds to where the curves intersect, which occurs at a point (0-dimensional). **(B)** With three adjustable ion channels (right), the solution for a single property is a surface (2-dimensional); hence, the joint solution for both properties corresponds to a curve (1-dimensional). Please note the connection with overdetermination and underdetermination discussed in Figure 4. Modified from Figure 5 of Yang et al. (2022).

Beyond considering the many ion channels expressed in a neuron, one must also consider the properties other than excitability that the neuron regulates. It is helpful to consider this problem in abstract terms before getting into biological details. The set of ion channel combinations able to produce the target value for two regulated properties corresponds to where solution manifolds for each property intersect (Yang et al., 2022; Figure 6). Unless the manifolds for each property are equivalent, the intersection is necessarily lower dimensional than the component manifolds: for example, two curves (one-dimensional, or 1-D) intersect at a point (0-dimensional), two planes (2-dimensional) intersect at a curve (1-dimensional), two volumes (3-dimensional) intersect as a plane (2-dimensional), etc. Recall that dimensionality of the manifold also reflects the number of adjustable ion channels (see above). The degeneracy of the joint solution thus corresponds to the number of adjustable ion channels minus the number of co-regulated properties. By extension, greater ion channel diversity is required to co-regulate multiple properties than is required to regulate any one property even if the same channels are shared across negative feedback loops.

**FIGURE 7:**
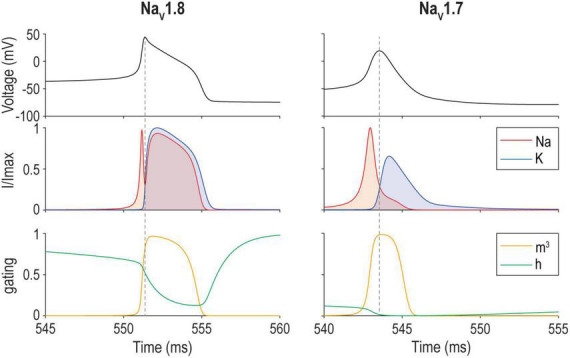
Energy efficiency of spike generation depends on overlap between Na^+^ and K^+^ currents. Sample spike evoked by a 17 pA current step applied to a model of a mouse nociceptive sensory neuron that relies on either Na_V_1.8 **(left)** or Na_V_1.7 **(right)**. The overlap between Na^+^ and K^+^ currents **(middle)** corresponds to the amount of “waste” current. Differences in activation (*m*) and inactivation (*h*) **(bottom)** explain differences in the spike waveform and energy efficiency. Na_V_1.8 and Na_V_1.7 models correspond to models for day *in vitro* 0 and 4–7, respectively, from Xie et al. (2022).

How do other cellular properties relate to excitability? Importantly, fast synaptic transmission and spike generation involve transmembrane ion flux down electrochemical gradients. Those gradients need to be replenished, not only to sustain synaptic transmission and spiking, but also to prevent secondary changes in osmotic pressure and cell volume. If sodium accumulates intracellularly, the neuron will swell and eventually rupture (Pasantes-Morales and Tuz, 2006). The human brain consumes about 20% of the body’s energy (Aiello and Wheeler, 1995), most of which is spent powering the Na^+^/K^+^-ATPase pump to maintain electrochemical gradients (Attwell and Laughlin, 2001). The pump removes three Na^+^ ions in exchange for two K^+^ ions per ATP. Metabolic costs (in ATP) thus depend on the total ion flux across the membrane. Action potentials are energetically expensive. The total energy cost obviously goes up with spike rate but the energy cost per spike also varies across cell types depending on their channel compositions (Sengupta et al., 2010). Energy cost per spike depends on the overlap in activation of Na^+^ and K^+^ channels (Hasenstaub et al., 2010; Figure 7). During depolarization, Na^+^ influx approaches the theoretical minimum required for charging the capacitance because the majority of K^+^ channels are not yet activated. During repolarization, however, Na^+^ current competes with K^+^ current until Na^+^ channels inactivate or until K^+^ current manages to repolarize the neuron enough to deactivate Na^+^ channels. The overlapping currents cancel each other and are wasted—like having a foot on the accelerator and the brake simultaneously—and thus determine the cost of an action potential (Sengupta et al., 2010).

Channel combinations yielding equivalent excitability may yield action potentials with very different energy efficiency (Yang et al., 2022), which may or may not be consequential for the neuron depending on its energy constraints. One might assume that spike generation should be as efficient as possible, but increasing efficiency has repercussions for safety factor, maximal firing rate, and signal-to-noise ratio (see Box 1). The appropriate balance to satisfy these competing interests will differ between neuron types, between different compartments of the same neuron (e.g., axon vs. soma), and between conditions (e.g., whether or not energy is limited, either by low supply or high demand). Determining if/how various properties are monitored is difficult without knowing what the error signal represents, which requires knowing how the error signal is encoded. That said, mitochondria produce the majority of ATP and are implicated in epilepsy (Zsurka and Kunz, 2015) and neurodegenerative disorders (Błaszczyk, 2020; Muddapu et al., 2020). Styr et al. (2019) recently identified a mitochondrial enzyme that regulates the firing rate set point in hippocampal circuits, thus linking energy regulation with excitability regulation (see also Ruggiero et al., 2021).

**BOX 1 Trade-offs between energy efficiency and performance.**

Natural selection balances costs and benefits; for example, the elongated tail of the male widowbird is advantageous for mating but disadvantageous for flying and hiding from predators (Andersson, 1982). Likewise, the encephalized human brain is more sophisticated but also costly such that the gut size became smaller to save energy (Aiello and Wheeler, 1995). Below we discuss how signal-to-noise ratio (SNR) and bandwidth are inversely correlated with energy efficiency.

*Signal-to-noise ratio.* Noise generally has detrimental effects on performance, notwithstanding many exceptions not discussed here. The ratio between the power of signal and background noise (i.e., SNR) is often used as a measure of information capacity (de Ruyter van Steveninck and Laughlin, 1996). Ion channels are inherently noisy due to random fluctuations between closed and open states. Given that the single-channel conductance is orders of magnitude smaller than the total conductance, a large number of channels may seem sufficient to increase SNR at the cellular level. However, channel noise decreases proportionally to the square root of the number of channels (White et al., 2000); in other words, to increase SNR by a factor of two, a neuron needs to quadruple the number of channels, not to mention the number of extra ATP-driven pumps and the associated metabolic costs. In fact, the cell volume physically limits the maximum number of channels, since approximately 0.11 μm^3^ of mitochondria is required per channel, given that one Na^+^ channel consumes 46 ATP/ms and mitochondria produce 400 ATP/ms per μm^3^ (Sengupta et al., 2013). On the other hand, channel noise places a lower limit on the number of channels and thus cell size. Smaller compartments need fewer channels to charge the total capacitance but are noisier; in fact, the rate of spontaneous action potentials increases exponentially below a critical diameter of 0.1 μm (Faisal et al., 2005).

*Bandwidth.* To optimize energy efficiency, the overlap between Na^+^ and K^+^ must be minimized (see Figure 7). But waiting for Na^+^ channels to inactivate rather than activating K^+^ channels delays repolarization, thus decreasing the maximal firing rate and limiting bandwidth. A lower membrane resistance shortens the membrane time constant, which can increase firing rate, but with an energetic cost. This is well illustrated in blowfly photoreceptors, whose bandwidth depends on leak (Niven et al., 2003a) and non-inactivating delayed rectifier channels (Laughlin and Weckström, 1993). These cells act like an open faucet and require substantial Na^+^ current to depolarize the cell. On the other hand, fast-inactivating *Shaker* K^+^ channels selectively amplify graded potentials, maximizing bandwidth while spending the minimum energy possible (Niven et al., 2003a,b), to increase coding efficiency (Levy and Baxter, 1996; Balasubramanian et al., 2001). Loss of *Shaker* channels results in decreased bandwidth, which, in turn, is compensated by leak channels at the cost of energy (Niven et al., 2003b). Clearly, performance is prioritized over metabolic cost in blowfly photoreceptors, despite diminishing returns for metabolic cost (Niven et al., 2007). Likewise, medial superior olive neurons in the auditory brainstem have a high energy demand, prioritizing performance but saving energy whenever possible (Remme et al., 2018). By comparison, many other cell types seem to operate near maximal efficiency (Sengupta et al., 2010; Al-Basha and Prescott, 2019).

Energy-dependent replenishment of electrochemical gradients is critical not only for electrical signaling, but also for controlling osmotic forces and volume. The volume of intracellular and extracellular compartments is especially important for the brain since the incompressible fluid comprising these compartments is enclosed by the skull (Strange, 1993; Wilson and Mongin, 2018). Swelling of brain cells in stroke or traumatic brain injury is dangerous because it results in a compensatory decrease in blood volume (Monroe-Kellie doctrine; Hellas and Andrew, 2021). Unlike astrocytes, neurons do not express aquaporins, or water channels, and thus regulate osmolarity via facilitated diffusion (ion channels) and active or secondary active transport (ATP-powered pumps and co-transporters) (Wilson and Mongin, 2018). If the intracellular sodium load is excessive and/or sodium removal is compromised (e.g., because of energy deficits due to reduced blood flow), spreading depolarization will ensue as electrochemical gradients are compromised, followed by silencing of brain activity, or spreading depression (Hellas and Andrew, 2021).

As evident from the discussion above, no property is regulated in isolation. Neurobiologists have focused on homeostatic regulation of synaptic strength and excitability, but a more holistic view is important for understanding the broader context in which that regulation occurs, and how it might be constrained (Hartwell et al., 1999; Frere and Slutsky, 2018). However, taking a more holistic view quickly reveals knowledge gaps. Even if we know that a property is regulated, delineating the negative feedback loop (i.e., identifying the error signal, set point, and all adjustable ion channels) is daunting. Synaptic scaling and excitability regulation have been extensively modeled as a negative feedback loop involving Ca^2+^ (LeMasson et al., 1993; Liu et al., 1998; O’Leary et al., 2013, 2014) since intracellular Ca^2+^ levels are well suited to transducing electrical activity into biochemical signals that modulate transcription, translation, post-translational modifications, and trafficking (Flavell and Greenberg, 2008). But independent error signals are needed to limit crosstalk between feedback loops (Yang et al., 2022), implying that additional error signals are encoded by other means or that multiple error signals are multiplexed in different aspects of a calcium signal. Needless to say, much more research is needed to rectify these knowledge gaps.

## Different ways homeostatic regulation can fail

Under normal conditions, if a homeostatically regulated system is perturbed, negative feedback will implement corrective changes that return the system’s output to the set point (see Figure 1). If a pathological change in excitability occurs (i.e., a neuron chronically fires too many or too few spikes), one must ask how that change occurred *despite* homeostatic regulation. This is often overlooked. A change in excitability is expected immediately after blocking an ion channel (or transiently increasing input; see Figure 2), but chronic blockade of the same channel will trigger myriad compensatory changes that are important in the longer term. This suggests that neurological disorders involving a chronic increase or decrease in neuronal excitability reflect a problem in homeostatic regulation rather than a problem with any one ion channel (Ratté and Prescott, 2016). Loss- or gain-of-function mutations in channels like Na_*V*_1.7, which cause congenital insensitivity to pain or painful neuropathies, respectively, seem to provide persuasive counterarguments, but when one digs deeper, the inconsistencies support rather than disprove the role of homeostatic regulation (Xie et al., 2022). Below, we discuss the different ways homeostatic regulation of excitability can fail.

An obvious reason for a regulated property to deviate from its set point is that negative feedback is overwhelmed, either because the perturbation is too strong or the negative feedback is too weak. In heat stroke, for example, prolonged exposure to high temperature and humidity triggers sweating but this is insufficient to maintain body temperature at 37°C (and sweating may eventually fail outright due to dehydration). The solution is to (1) remove or at least reduce the perturbation, (2) strengthen the negative feedback, and/or (3) support the negative feedback with an exogenous intervention. In the case of heat stroke, this would involve (1) moving into the shade to reduce heating, (2) drinking fluids to support sweating, and (3) actively cooling with wet towels or a cool bath.

Alternatively, the set point may be altered so that the regulated property is maintained but at the “wrong” set point. An example is fever, where the body deliberately increases its temperature to a new set point > 37°C, usually because of infection although there are other causes (e.g., inflammation, neoplasm, or even head injury). The best treatment is an antipyretic drug, which, at least in the case of anti-inflammatories (e.g., ibuprofen), act by blocking signals that maintain the wrong (increased) set point, thus restoring the set point to a normal value. Whereas active cooling is very effective against heat stroke, it is less effective against fever because it is working *against* endogenous thermoregulatory feedback mechanisms rather than supporting them. The more robust that negative feedback is, the harder it is to fight against.

If excitability is robustly regulated thanks to degeneracy, then its homeostatic regulation is unlikely to be overwhelmed by a pathological change in a specific ion channel or some other parameter (like in heat stroke); and if it is overwhelmed, then supporting that regulation (like with active cooling) should be effective in restoring normal excitability. But if excitability is being misregulated to the wrong set point (like in fever) and if degeneracy renders that homeostatic regulation very robust, then restoring normal excitability by fighting against that regulation may be a losing battle (Ratté and Prescott, 2016). This might explain why chronic changes in neuronal excitability are relatively rare, but when they do occur, they tend to be difficult to treat—because the problem is with the set point rather than some other aspect of the feedback loop. For instance, in neuropathic pain, which is pain caused by damage to the nervous system, current analgesics provide significant pain relief in only 1 in 5 patients (Moulin et al., 2015). Likewise, about 1 in 3 epilepsy patients suffers from drug-resistant epilepsy (Kalilani et al., 2018; Sultana et al., 2021), defined as failure of at least two appropriately used antiepileptic drug regimens to prevent seizures. Rather than fighting against intact but misguided regulation, one would ideally restore the set point to its proper value (like taking antipyretics to reduce fever). This requires deeper understanding of set points, which is why the study by Styr et al. (2019) on the regulation of firing rate set point is notable. More generally, the idea of targeting therapies to homeostatic regulation has started to gain traction (Kavalali and Monteggia, 2023). Epilepsy and other conditions like tinnitus, or ringing in the ears, have been linked to problems in homeostatic regulation (Yang et al., 2011; Yang and Bao, 2013; Lignani et al., 2020; Issa et al., 2023). The negative regulation of homeostatic regulation by the PARbZIP family of transcription factors, which helps prevent seizures (Valakh et al., 2023), is also notable.

One must also consider potential problems with the error signal. For example, a single thermostat located on the ground floor of a house may not yield the desired temperature throughout the house even if the target temperature is correctly set and the negative feedback is functioning properly (on the ground floor). The problem is that the error signal does not capture disturbances in the upstairs air temperature. This constitutes an alignment problem (Christian, 2020). In the context of regulating excitability, intracellular calcium level is only a proxy for spike rate, meaning changes in the amount of calcium entry per spike or myriad other changes might distort the relationship between the error signal and firing rate. Hence, negative feedback might reduce the error signal to zero without actually restoring firing rate to its intended value. Crosstalk between the error signals used by different feedback loops could also be problematic.

**FIGURE 8:**
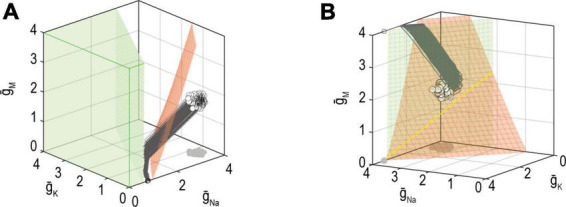
Mechanisms for failing to co-regulate two properties. **(A)** If the solution manifold for firing rate (red) does not intersect the solution manifold for energy efficiency (green), then no ion channel combinations exist that achieve the target for firing rate *and* energy efficiency. In other words, solutions for each property exist but are incompatible with one another. In this example, homeostatic regulation found an intermediate, compromise solution. **(B)** Solution manifolds for different properties may exist and intersect, yet ion channel densities may not reach the joint solution (yellow) because of conflicting adjustments arising from different error signals. Different feedback loops cause solutions to approach the manifold with different trajectories, which may be incompatible. Modified from Figures 9 and 10 of Yang et al. (2022).

The failure mechanisms discussed above consider regulation of a single property but additional failure mechanisms are possible when multiple properties are co-regulated. Firstly, as explained in Figure 6, the joint solution for two properties corresponds to the intersection of the solution manifolds for each property. However, even if a large solution manifold exists for each property, the two manifolds might not intersect (Figure 8A); in other words, ion channel combinations that produce the target value for one or the other property exist, but there are none that produce the target values for *both* properties (Yang et al., 2022). If one property has stronger feedback than the other, then the more strongly regulated property will be maintained at the expense of the other. Alternatively, neither property might reach its set point, with solutions instead settling on some compromise that balances the error signal from each loop, as seen in Figure 8A (Jedlicka et al., 2022).

The other possibility is that a joint solution exists but cannot be found. In other words, the solution manifolds for each property intersect, but converging on that intersection may be difficult (Figure 8B). Consider that each error signal may try to adjust expression of the same channels in opposite directions. Depending on the relative rates with which ion channels are being co-adjusted by each feedback loop, the adjustments imposed by each loop (evident as trajectories on the graph) may be incompatible. Similarly, if two feedback loops use the same error signal (e.g., calcium) but have unequal set points, the feedback will “windup” rather than settling on a joint solution (O’Leary et al., 2014).

An emerging theme is that solutions, adjustments, error signals, etc., must be compatible across feedback loops when co-regulating multiple properties. Such incompatibilities are absent from simulations that only model one feedback loop. In contrast, experimental analysis of a feedback loop of interest might be severely compromised by the ongoing effects of an unidentified feedback loop. As already alluded to, unknown unknowns can be problematic but must be grappled with to develop a more integrative understanding of homeostasis. Reciprocal interactions between properties have been studied in Alzheimer’s disease (Frere and Slutsky, 2018; Styr and Slutsky, 2018), Parkinson’s disease (Büeler, 2009; Haddad and Nakamura, 2015) and epilepsy (Sharma and Prasad, 2017), which is a step in the right direction.

## Discussion

In this review, we defined key concepts including degeneracy, pleiotropy, and functional overlap, and we linked those concepts to the mapping between parameters and properties. Degeneracy enables different parameter (channel) combinations to convey equivalent output (excitability). This increases the robustness of homeostatic regulation by allowing compensatory changes to distribute across many parameters. This ability to reconfigure solutions is critical when dealing with pleiotropic channels because adjusting a pleiotropic channel to help regulate one property risks disrupting other properties. Degeneracy helps ensure there are many degrees of freedom so that a solution yielding the intended value for all regulated properties can be found. That said, homeostatic regulation can nevertheless fail because a perturbation is too strong or feedback is too weak, or it might fail to give the intended outcome not because regulation failed, but because it succeeded in regulating the system to a pathologically disturbed set point. Co-regulating multiple properties introduces additional complications that boil down to incompatibilities between feedback loops. Despite the many challenges, unraveling the intersecting feedback loops responsible for homeostatic regulation of neuronal function is rewarding in its own right, but might also reveal key insights into chronic neurological disorders that continue to elude treatment.

## Author contributions

Both authors wrote and edited the manuscript, contributed to the article, and approved the submitted version.

## References

[B1] Aiello L. C. Wheeler P. (1995). The expensive-tissue hypothesis: The brain and the digestive system in human and primate evolution. *Curr. Anthropol.* 36 199–221.

[B2] Al-Basha D. Prescott S. A. (2019). Intermittent failure of spike propagation in primary afferent neurons during tactile stimulation. *J. Neurosci*. 39 9927–9939. 10.1523/jneurosci.0975-19.2019 31672792 PMC6978946

[B3] Amendola J. Woodhouse A. Martin-Eauclaire M.-F. Goaillard J.-M. (2012). Ca^2+^/cAMP-sensitive covariation of I_*A*_ and I_*H*_ voltage dependences tunes rebound firing in dopaminergic neurons. *J. Neurosci.* 32 2166–2181. 10.1523/JNEUROSCI.5297-11.2012 22323729 PMC6621702

[B4] Andersson M. (1982). Female choice selects for extreme tail length in a widowbird. *Nature* 299 818–820.

[B5] Attwell D. Laughlin S. B. (2001). An energy budget for signaling in the grey matter of the brain. *J. Cereb. Blood. Flow. Metab*. 21 1133–1145. 10.1097/00004647-200110000-00001 11598490

[B6] Baines R. A. (2005). Neuronal homeostasis through translational control. *Mol. Neurobiol.* 32 113–121. 10.1385/MN:32:2:113 16215276

[B7] Baines R. A. Uhler J. P. Thompson A. Sweeney S. T. Bate M. (2001). Altered electrical properties in Drosophila neurons developing without synaptic transmission. *J. Neurosci.* 21 1523–1531. 10.1523/jneurosci.21-05-01523.2001 11222642 PMC6762927

[B8] Balasubramanian V. Kimber D. Berry M. J. II. (2001). Metabolically efficient information processing. *Neural Comput.* 13 799–815. 10.1162/089976601300014358 11255570

[B9] Barnes S. J. Franzoni E. Jacobsen R. I. Erdelyi F. Szabo G. Clopath C. (2017). Deprivation-induced homeostatic spine scaling in vivo is localized to dendritic branches that have undergone recent spine loss. *Neuron* 96 871–882.e5. 10.1016/j.neuron.2017.09.052 29107520 PMC5697914

[B10] Bennett D. L. Clark A. J. Huang J. Waxman S. G. Dib-Hajj S. D. (2019). The role of voltage-gated sodium channels in pain signaling. *Physiol. Rev.* 99 1079–1151. 10.1152/physrev.00052.2017 30672368

[B11] Billman G. E. (2020). Homeostasis: The underappreciated and far too often ignored central organizing principle of physiology. *Front. Physiol.* 11:200. 10.3389/fphys.2020.00200 32210840 PMC7076167

[B12] Błaszczyk J. W. (2020). Energy metabolism decline in the aging brain—pathogenesis of neurodegenerative disorders. *Metabolites* 10:450. 10.3390/metabo10110450 33171879 PMC7695180

[B13] Brickley S. G. Revilla V. Cull-Candy S. G. Wisden W. Farrant M. (2001). Adaptive regulation of neuronal excitability by a voltage-independent potassium conductance. *Nature* 409 88–92. 10.1038/35051086 11343119

[B14] Büeler H. (2009). Impaired mitochondrial dynamics and function in the pathogenesis of Parkinson’s disease. *Exp. Neurol.* 218 235–246. 10.1016/j.expneurol.2009.03.006 19303005

[B15] Burrone J. O’Byrne M. Murthy V. N. (2002). Multiple forms of synaptic plasticity triggered by selective suppression of activity in individual neurons. *Nature* 420 414–418. 10.1038/nature01242 12459783

[B16] Christian B. (2020). *The alignment problem: Machine learning and human values.* New York, NY: W.W. Norton & Company.

[B17] Davis G. W. (2013). Homeostatic signaling and the stabilization of neural function. *Neuron* 80 718–728. 10.1016/j.neuron.2013.09.044 24183022 PMC3856728

[B18] Davis G. W. Bezprozvanny I. (2001). Maintaining the stability of neural function: A homeostatic hypothesis. *Annu. Rev. Physiol.* 63 847–869. 10.1146/annurev.physiol.63.1.847 11181978

[B19] Davis G. W. Goodman C. S. (1998). Synapse-specific control of synaptic efficacy at the terminals of a single neuron. *Nature* 392 82–86. 10.1038/32176 9510251

[B20] Day T. A. (2005). Defining stress as a prelude to mapping its neurocircuitry: No help from allostasis. *Prog. Neuropsychopharmacol. Biol. Psychiatry* 29 1195–1200. 10.1016/j.pnpbp.2005.08.005 16213079

[B21] de Ruyter van Steveninck R. R. Laughlin S. B. (1996). The rate of information transfer at graded-potential synapses. *Nature* 379 642–645. 10.1038/379642a0

[B22] Desai N. S. Cudmore R. H. Nelson S. B. Turrigiano G. G. (2002). Critical periods for experience-dependent synaptic scaling in visual cortex. *Nat. Neurosci.* 5 783–789. 10.1038/nn878 12080341

[B23] Desai N. S. Rutherford L. C. Turrigiano G. G. (1999). Plasticity in the intrinsic excitability of cortical pyramidal neurons. *Nat. Neurosci.* 2 515–520. 10.1038/9165 10448215

[B24] Edelman G. M. Gally J. A. (2001). Degeneracy and complexity in biological systems. *Proc. Natl. Acad. Sci. U. S. A.* 98 13763–13768. 10.1073/pnas.231499798 11698650 PMC61115

[B25] Faisal A. A. White J. A. Laughlin S. B. (2005). Ion-channel noise places limits on the miniaturization of the brain’s wiring. *Curr. Biol.* 15 1143–1149. 10.1016/j.cub.2005.05.056 15964281

[B26] Flavell S. W. Greenberg M. E. (2008). Signaling mechanisms linking neuronal activity to gene expression and plasticity of the nervous system. *Annu. Rev. Neurosci.* 31 563–590. 10.1146/annurev.neuro.31.060407.125631 18558867 PMC2728073

[B27] Franci A. O’Leary T. Golowasch J. (2020). Positive dynamical networks in neuronal regulation: How tunable variability coexists with robustness. *IEEE Control Syst. Lett.* 4 946–951. 10.1109/LCSYS.2020.2997214

[B28] Frere S. Slutsky I. (2018). Alzheimer’s disease: From firing instability to homeostasis network collapse. *Neuron* 9 32–58. 10.1016/j.neuron.2017.11.028 29301104

[B29] Gainey M. A. Aman J. W. Feldman D. E. (2018). Rapid disinhibition by adjustment of PV intrinsic excitability during whisker map plasticity in mouse S1. *J. Neurosci.* 38 4749–4761. 10.1523/JNEUROSCI.3628-17.2018 29678876 PMC5956988

[B30] Gainey M. A. Feldman D. E. (2017). Multiple shared mechanisms for homeostatic plasticity in rodent somatosensory and visual cortex. *Philos. Trans. R. Soc. Lond. B Biol. Sci.* 372:20160157. 10.1098/rstb.2016.0157 28093551 PMC5247589

[B31] Gaiteri C. Ding Y. French B. Tseng G. Sibille E. (2014). Beyond modules and hubs: The potential of gene coexpression networks for investigating molecular mechanisms of complex brain disorders. *Genes Brain Behav.* 13 13–24. 10.1111/gbb.12106 24320616 PMC3896950

[B32] Galante M. Nistri A. Ballerini L. (2000). Opposite changes in synaptic activity of organotypic rat spinal cord cultures after chronic block of AMPA/kainate or glycine and GABA_*A*_ receptors. *J. Physiol.* 523 639–651. 10.1111/j.1469-7793.2000.t01-1-00639.x 10718744 PMC2269832

[B33] Goaillard J.-M. Marder E. (2021). Ion channel degeneracy, variability, and covariation in neuron and circuit resilience. *Annu. Rev. Neurosci.* 44 335–357. 10.1146/annurev-neuro-092920-121538 33770451

[B34] Goel A. Lee H.-K. (2007). Persistence of experience-induced homeostatic synaptic plasticity through adulthood in superficial layers of mouse visual cortex. *J. Neurosci.* 27 6692–6700. 10.1523/JNEUROSCI.5038-06.2007 17581956 PMC2601561

[B35] Golowasch J. Abbott L. F. Marder E. (1999). Activity-dependent regulation of potassium currents in an identified neuron of the stomatogastric ganglion of the crab *Cancer borealis*. *J. Neurosci.* 19:RC33. 10.1523/JNEUROSCI.19-20-j0004.1999 10516335 PMC6782763

[B36] Golowasch J. Goldman M. S. Abbott L. F. Marder E. (2002). Failure of averaging in the construction of a conductance-based neuron model. *J. Neurophysiol.* 87 1129–1131. 10.1152/jn.00412.2001 11826077

[B37] Greenhill S. D. Ranson A. Fox K. (2015). Hebbian and homeostatic plasticity mechanisms in regular spiking and intrinsic bursting cells of cortical layer 5. *Neuron* 88 539–552. 10.1016/j.neuron.2015.09.025 26481037 PMC4643308

[B38] Grubb M. S. Burrone J. (2010). Activity-dependent relocation of the axon initial segment fine-tunes neuronal excitability. *Nature* 465 1070–1074. 10.1038/nature09160 20543823 PMC3196626

[B39] Haddad D. Nakamura K. (2015). Understanding the susceptibility of dopamine neurons to mitochondrial stressors in Parkinson’s disease. *FEBS Lett.* 589 3702–3713. 10.1016/j.febslet.2015.10.021 26526613 PMC4679488

[B40] Hartwell L. H. Hopfield J. J. Leibler S. Murray A. W. (1999). From molecular to modular cell biology. *Nature* 402 C47–C52. 10.1038/35011540 10591225

[B41] Hasenstaub A. Otte S. Callaway E. Sejnowski T. J. (2010). Metabolic cost as a unifying principle governing neuronal biophysics. *Proc. Natl. Acad. Sci. U. S. A*. 107 12329–12334. 10.1073/pnas.0914886107 20616090 PMC2901447

[B42] Hellas J. A. Andrew R. D. (2021). Neuronal swelling: A non-osmotic consequence of spreading depolarization. *Neurocrit. Care* 35 112–134. 10.1007/s12028-021-01326-w 34498208 PMC8536653

[B43] Hudson A. E. Prinz A. A. (2010). Conductance ratios and cellular identity. *PLoS Comput. Biol*. 6:e1000838. 10.1371/journal.pcbi.1000838 20628472 PMC2895636

[B44] Issa N. P. Nunn K. C. Wu S. Haider H. A. Tao J. X. (2023). Putative roles for homeostatic plasticity in epileptogenesis. *Epilepsia* 64 539–552. 10.1111/epi.17500 36617338 PMC10015501

[B45] Jain A. Narayanan R. (2020). Degeneracy in the emergence of spike-triggered average of hippocampal pyramidal neurons. *Sci. Rep*. 10:374. 10.1038/s41598-019-57243-8 31941985 PMC6962224

[B46] Jedlicka P. Bird A. Cuntz H. (2022). Pareto optimality, economy-effectiveness trade-offs and ion channel degeneracy: Improving population models of neurons. *Open Biol.* 12:220073. 10.1098/rsob.220073 35857898 PMC9277232

[B47] Kalilani L. Sun X. Pelgrims B. Noack-Rink M. Villanueva V. (2018). The epidemiology of drug-resistant epilepsy: A systematic review and meta-analysis. *Epilepsia* 59 2179–2193. 10.1111/epi.14596 30426482

[B48] Kavalali E. T. Monteggia L. M. (2023). Rapid homeostatic plasticity and neuropsychiatric therapeutics. *Neuropsychopharmacology* 48 54–60. 10.1038/s41386-022-01411-4 35995973 PMC9700859

[B49] Keck T. Hübener M. Bonhoeffer T. (2017). Interactions between synaptic homeostatic mechanisms: An attempt to reconcile BCM theory, synaptic scaling, and changing excitation/inhibition balance. *Curr. Opin. Neurobiol.* 43 87–93. 10.1016/j.conb.2017.02.003 28236778

[B50] Khorkova O. Golowasch J. (2007). Neuromodulators, not activity, control coordinated expression of ionic currents. *J. Neurosci*. 27 8709–8718. 10.1523/JNEUROSCI.1274-07.2007 17687048 PMC3558984

[B51] Kilman V. van Rossum M. C. W. Turrigiano G. G. (2002). Activity deprivation reduces miniature IPSC amplitude by decreasing the number of postsynaptic GABA_*A*_ receptors clustered at neocortical synapses. *J. Neurosci.* 22 1328–1337. 10.1523/JNEUROSCI.22-04-01328.2002 11850460 PMC6757564

[B52] Kirkwood A. Rioult M. G. Bear M. F. (1996). Experience-dependent modification of synaptic plasticity in visual cortex. *Nature* 381 526–528. 10.1038/381526a0 8632826

[B53] Kodama T. Gittis A. H. Shin M. Kelleher K. Kolkman K. E. McElvain L. (2020). Graded coexpression of ion channel, neurofilament, and synaptic genes in fast-spiking vestibular nucleus neurons. *J. Neurosci.* 40 496–508. 10.1523/JNEUROSCI.1500-19.2019 31719168 PMC6961989

[B54] Kuba H. Oichi Y. Ohmori H. (2010). Presynaptic activity regulates Na^+^ channel distribution at the axon initial segment. *Nature* 465 1075–1078. 10.1038/nature09087 20543825

[B55] Laughlin S. B. Weckström M. (1993). Fast and slow photoreceptors — a comparative study of the functional diversity of coding and conductances in the Diptera. *J. Comp. Physiol. A* 172 593–609. 10.1007/BF00213682

[B56] Lee H.-K. Kirkwood A. (2019). Mechanisms of homeostatic synaptic plasticity in vivo. *Front. Cell. Neurosci.* 13:520. 10.3389/fncel.2019.00520 31849610 PMC6901705

[B57] LeMasson G. Marder E. Abbott L. F. (1993). Activity-dependent regulation of conductances in model neurons. *Science* 259 1915–1917. 10.1126/science.8456317 8456317

[B58] Levy W. B. Baxter R. A. (1996). Energy efficient neural codes. *Neural Comput*. 8 531–543. 10.1162/neco.1996.8.3.531 8868566

[B59] Lignani G. Baldelli P. Marra V. (2020). Homeostatic plasticity in epilepsy. *Front. Cell. Neurosci.* 14:197. 10.3389/fncel.2020.00197 32676011 PMC7333442

[B60] Liu Z. Golowasch J. Marder E. Abbott L. F. (1998). A model neuron with activity-dependent conductances regulated by multiple calcium sensors. *J. Neurosci*. 18 2309–2320. 10.1523/JNEUROSCI.18-07-02309.1998 9502792 PMC6793093

[B61] Marco A. Konikoff C. Karr T. L. Kumar S. (2009). Relationship between gene co-expression and sharing of transcription factor binding sites in Drosophila melanogaster. *Bioinformatics* 25 2473–2477. 10.1093/bioinformatics/btp462 19633094 PMC2752616

[B62] Mee C. J. Pym E. C. G. Moffat K. G. Baines R. A. (2004). Regulation of neuronal excitability through pumilio-dependent control of a sodium channel gene. *J. Neurosci.* 24 8695–8703. 10.1523/JNEUROSCI.2282-04.2004 15470135 PMC6729971

[B63] Mishra P. Narayanan R. (2021). Stable continual learning through structured multiscale plasticity manifolds. *Curr. Opin. Neurobiol.* 70 51–63. 10.1016/j.conb.2021.07.009 34416674 PMC7611638

[B64] Moulin D. E. Clark A. J. Gordon A. Lynch M. Morley-Forster P. K. Nathan H. (2015). Long-term outcome of the management of chronic neuropathic pain: A prospective observational study. *J. Pain* 16 852–861. 10.1016/j.jpain.2015.05.011 26080044

[B65] Muddapu V. R. Darshini S. A. P. Chakravarthy V. S. Gromiha M. M. (2020). Neurodegenerative diseases – is metabolic deficiency the root cause?. *Front. Neurosci.* 14:213. 10.3389/fnins.2020.00213 32296300 PMC7137637

[B66] Niven J. E. Anderson J. C. Laughlin S. B. (2007). Fly photoreceptors demonstrate energy-information trade-offs in neural coding. *PLoS Biol.* 5:e116. 10.1371/journal.pbio.0050116 17373859 PMC1828148

[B67] Niven J. E. Vahasoyrinki M. Kauranen M. Hardie R. C. Juusola M. Weckstrom M. (2003a). The contribution of Shaker K^+^ channels to the information capacity of Drosophila photoreceptors. *Nature* 421 630–634. 10.1038/nature01384 12571596

[B68] Niven J. E. Vähäsöyrinki M. Juusola M. (2003b). Shaker K^+^-channels are predicted to reduce the metabolic cost of neural information in Drosophila photoreceptors. *Proc. Biol. Sci.* 270 S58–S61. 10.1098/rsbl.2003.0010 12952637 PMC1698034

[B69] O’Brien R. J. Kamboj S. Ehlers M. D. Rosen K. R. Fischbach G. D. Huganir R. L. (1998). Activity-dependent modulation of synaptic AMPA receptor accumulation. *Neuron* 21 1067–1078. 10.1016/s0896-6273(00)80624-8 9856462

[B70] O’Leary T. van Rossum M. C. W. Wyllie D. J. A. (2010). Homeostasis of intrinsic excitability in hippocampal neurones: Dynamics and mechanism of the response to chronic depolarization. *J. Physiol.* 588 157–170. 10.1113/jphysiol.2009.181024 19917565 PMC2821556

[B71] O’Leary T. Williams A. H. Caplan J. S. Marder E. (2013). Correlations in ion channel expression emerge from homeostatic tuning rules. *Proc. Natl. Acad. Sci. U. S. A.* 110 E2645–E2654. 10.1073/pnas.1309966110 23798391 PMC3710808

[B72] O’Leary T. Williams A. H. Franci A. Marder E. (2014). Cell types, network homeostasis, and pathological compensation from a biologically plausible ion channel expression model. *Neuron* 82 809–821. 10.1016/j.neuron.2014.04.002 24853940 PMC4109293

[B73] O’Leary T. Wyllie D. J. A. (2011). Neuronal homeostasis: Time for a change?. *J. Physiol.* 589 4811–4826. 10.1113/jphysiol.2011.210179 21825033 PMC3224876

[B74] Olypher A. V. Calabrese R. L. (2007). Using constraints on neuronal activity to reveal compensatory changes in neuronal parameters. *J. Neurophysiol.* 98 3749–3758. 10.1152/jn.00842.2007 17855581

[B75] Pandey A. Hardingham N. Fox K. (2022). Differentiation of Hebbian and homeostatic plasticity mechanisms within layer 5 visual cortex neurons. *Cell Rep.* 39:110892. 10.1016/j.celrep.2022.110892 35649371 PMC9637998

[B76] Pan-Vazquez A. Wefelmeyer W. Sabater V. G. Neves G. Burrone J. (2020). Activity-dependent plasticity of axo-axonic synapses at the axon initial segment. *Neuron* 106 265–276.e6. 10.1016/j.neuron.2020.01.037 32109363 PMC7181187

[B77] Pasantes-Morales H. Tuz K. (2006). Volume changes in neurons: Hyperexcitability and neuronal death. *Contrib. Nephrol.* 152 221–240. 10.1159/000096326 17065815

[B78] Ratté S. Prescott S. A. (2016). Afferent hyperexcitability in neuropathic pain and the inconvenient truth about its degeneracy. *Curr. Opin. Neurobiol.* 36 31–37. 10.1016/j.conb.2015.08.007 26363576

[B79] Remme M. W. H. Rinzel J. Schreiber S. (2018). Function and energy consumption constrain neuronal biophysics in a canonical computation: Coincidence detection. *PLoS Comput. Biol.* 14:e1006612. 10.1371/journal.pcbi.1006612 30521528 PMC6312336

[B80] Richter J. D. Bassell G. J. Klann E. (2015). Dysregulation and restoration of translational homeostasis in fragile X syndrome. *Nat. Rev. Neurosci.* 16 595–605. 10.1038/nrn4001 26350240 PMC4688896

[B81] Ruggiero A. Katsenelson M. Slutsky I. (2021). Mitochondria: New players in homeostatic regulation of firing rate set points. *Trends Neurosci.* 44 605–618. 10.1016/j.tins.2021.03.002 33865626

[B82] Santin J. M. Schulz D. J. (2019). Membrane voltage is a direct feedback signal that influences correlated ion channel expression in neurons. *Curr. Biol.* 29 1683–1688.e2. 10.1016/j.cub.2019.04.008 31080077 PMC6677403

[B83] Schneider M. Bird A. D. Gidon A. Triesch J. Jedlicka P. Cuntz H. (2022). Biological complexity facilitates tuning of the neuronal parameter space. *bioRxiv* [Preprint]. 10.1101/2021.05.04.442120 PMC10353791 37399220

[B84] Schulz D. J. Goaillard J. M. Marder E. E. (2007). Quantitative expression profiling of identified neurons reveals cell-specific constraints on highly variable levels of gene expression. *Proc. Natl. Acad. Sci. U. S. A.* 104 13187–13191. 10.1073/pnas.0705827104 17652510 PMC1933263

[B85] Sengupta B. Faisal A. A. Laughlin S. B. Niven J. E. (2013). The effect of cell size and channel density on neuronal information encoding and energy efficiency. *J. Cereb. Blood Flow Metab.* 33 1465–1473. 10.1038/jcbfm.2013.103 23778164 PMC3764378

[B86] Sengupta B. Stemmler M. Laughlin S. B. Niven J. E. (2010). Action potential energy efficiency varies among neuron types in vertebrates and invertebrates. *PLoS Comput. Biol*. 6:e1000840. 10.1371/journal.pcbi.1000840 20617202 PMC2895638

[B87] Shao Y. R. Isett B. R. Miyashita T. Chung J. Pourzia O. Gasperini R. J. (2013). Plasticity of recurrent L2/3 inhibition and gamma oscillations by whisker experience. *Neuron* 80 210–222. 10.1016/j.neuron.2013.07.026 24094112 PMC3792397

[B88] Sharma S. Prasad A. N. (2017). Inborn errors of metabolism and epilepsy: Current understanding, diagnosis, and treatment approaches. *Int. J. Mol. Sci.* 18:1384. 10.3390/ijms18071384 28671587 PMC5535877

[B89] Soofi W. Archila S. Prinz A. A. (2012). Co-variation of ionic conductances supports phase maintenance in stomatogastric neurons. *J. Comput. Neurosci*. 33 77–95. 10.1007/s10827-011-0375-3 22134522 PMC3394871

[B90] Strange K. (1993). Maintenance of cell volume in the central nervous system. *Pediatr. Nephrol.* 7 689–697. 10.1007/BF00852580 8251345

[B91] Styr B. Gonen N. Zarhin D. Ruggiero A. Atsmon R. Gazit N. (2019). Mitochondrial regulation of the hippocampal firing rate set point and seizure susceptibility. *Neuron* 102 1009–1024.e8. 10.1016/j.neuron.2019.03.045 31047779 PMC6559804

[B92] Styr B. Slutsky I. (2018). Imbalance between firing homeostasis and synaptic plasticity drives early-phase Alzheimer’s disease. *Nat. Neurosci.* 21 463–473. 10.1038/s41593-018-0080-x 29403035 PMC6533171

[B93] Sultana B. Panzini M. Carpentier A. V. Comtois J. Rioux B. Gore G. (2021). Incidence and prevalence of drug-resistant epilepsy: A systematic review and meta-analysis. *Neurology* 96 805–817. 10.1212/WNL.0000000000011839 33722992

[B94] Swensen A. M. Bean B. P. (2005). Robustness of burst firing in dissociated purkinje neurons with acute or long-term reductions in sodium conductance. *J. Neurosci.* 25 3509–3520. 10.1523/JNEUROSCI.3929-04.2005 15814781 PMC6725377

[B95] Tapia M. Baudot P. Formisano-Tréziny C. Dufour M. A. Temporal S. Lasserre M. (2018). Neurotransmitter identity and electrophysiological phenotype are genetically coupled in midbrain dopaminergic neurons. *Sci. Rep.* 8:13637. 10.1038/s41598-018-31765-z 30206240 PMC6134142

[B96] Temporal S. Lett K. M. Schulz D. J. (2014). Activity-dependent feedback regulates correlated ion channel mRNA levels in single identified motor neurons. *Curr. Biol*. 24 1899–1904. 10.1016/j.cub.2014.06.067 25088555

[B97] Tien N.-W. Kerschensteiner D. (2018). Homeostatic plasticity in neural development. *Neural Dev.* 13:9. 10.1186/s13064-018-0105-x 29855353 PMC5984303

[B98] Tien N.-W. Soto F. Kerschensteiner D. (2017). Homeostatic plasticity shapes cell-type-specific wiring in the retina. *Neuron* 94 656–665.e4. 10.1016/j.neuron.2017.04.016 28457596 PMC5477664

[B99] Tobin A. E. Cruz-Bermudez N. D. Marder E. Schulz D. J. (2009). Correlations in ion channel mRNA in rhythmically active neurons. *PLoS One* 4:e6742. 10.1371/journal.pone.0006742 19707591 PMC2727049

[B100] Tran T. Unal C. T. Severin D. Zaborszky L. Rotstein H. G. Kirkwood A. (2019). Ionic current correlations are ubiquitous across phyla. *Sci. Rep.* 9:1687. 10.1038/s41598-018-38405-6 30737430 PMC6368568

[B101] Turrigiano G. (2011). Too many cooks? Intrinsic and synaptic homeostatic mechanisms in cortical circuit refinement. *Annu. Rev. Neurosci.* 34 89–103. 10.1146/annurev-neuro-060909-153238 21438687

[B102] Turrigiano G. (2012). Homeostatic synaptic plasticity: Local and global mechanisms for stabilizing neuronal function. *Cold Spring Harb. Perspect. Biol.* 4:a005736. 10.1101/cshperspect.a005736 22086977 PMC3249629

[B103] Turrigiano G. Abbott L. F. Marder E. (1994). Activity-dependent changes in the intrinsic properties of cultured neurons. *Science* 264 974–977. 10.1126/science.8178157 8178157

[B104] Turrigiano G. G. (2008). The self-tuning neuron: Synaptic scaling of excitatory synapses. *Cell* 135 422–435. 10.1016/j.cell.2008.10.008 18984155 PMC2834419

[B105] Turrigiano G. G. Leslie K. R. Desai N. S. Rutherford L. C. Nelson S. B. (1998). Activity-dependent scaling of quantal amplitude in neocortical neurons. *Nature* 391 892–896. 10.1038/36103 9495341

[B106] Turrigiano G. G. Nelson S. B. (2004). Homeostatic plasticity in the developing nervous system. *Nat. Rev. Neurosci.* 5 97–107. 10.1038/nrn1327 14735113

[B107] Valakh V. Wise D. Zhu X. A. Sha M. Fok J. Van Hooser S. D. (2023). A transcriptional constraint mechanism limits the homeostatic response to activation deprivation in mammalian neocortex. *Elife* 12:e74899. 10.7554/eLife.74899 36749029 PMC10010687

[B108] Wefelmeyer W. Puhl C. J. Burrone J. (2016). Homeostatic plasticity of subcellular neuronal structures: From inputs to outputs. *Trends Neurosci.* 39 656–667. 10.1016/j.tins.2016.08.004 27637565 PMC5236059

[B109] Wen W. Turrigiano G. G. (2021). Developmental regulation of homeostatic plasticity in mouse primary visual cortex. *J. Neurosci.* 41 9891–9905. 10.1523/JNEUROSCI.1200-21.2021 34686546 PMC8638690

[B110] White J. A. Rubinstein J. T. Kay A. R. (2000). Channel noise in neurons. *Trends Neurosci.* 23 131–137. 10.1016/s0166-2236(99)01521-0 10675918

[B111] Wilson C. S. Mongin A. A. (2018). Cell volume control in healthy brain and neuropathologies. *Curr. Top. Membr.* 81 385–455. 10.1016/bs.ctm.2018.07.006 30243438 PMC6416787

[B112] Xie Y.-F. Yang J. Ratté S. Prescott S. A. (2022). Equivalent excitability through different sodium channel subtypes and implications for analgesia by subtype-selective drugs. *bioRxiv* [Preprint]. 10.1101/2022.10.04.510784 PMC11060714 38687187

[B113] Yang J. Shakil H. Ratté S. Prescott S. A. (2022). Minimal requirements for a neuron to coregulate many properties and the implications for ion channel correlations and robustness. *Elife* 11:e72875. 10.7554/eLife.72875 35293858 PMC8986315

[B114] Yang S. Bao S. (2013). Homeostatic mechanisms and treatment of tinnitus. *Restor. Neurol. Neurosci.* 31 99–108. 10.3233/RNN-120248 23435453

[B115] Yang S. Weiner B. D. Zhang L. S. Cho S.-J. Bao S. (2011). Homeostatic plasticity drives tinnitus perception in an animal model. *Proc. Natl Acad. Sci. U. S. A.* 108 14974–14979. 10.1073/pnas.1107998108 21896771 PMC3169130

[B116] Zhang Z. Marro S. G. Zhang Y. Arendt K. L. Patzke C. Zhou B. (2018). The fragile X mutation impairs homeostatic plasticity in human neurons by blocking synaptic retinoic acid signaling. *Sci. Trans. Med.* 10:eaar4338. 10.1126/scitranslmed.aar4338 30068571 PMC6317709

[B117] Zhao S. Golowasch J. (2012). Ionic current correlations underlie the global tuning of large numbers of neuronal activity attributes. *J. Neurosci*. 32 13380–13388. 10.1523/jneurosci.6500-11.2012 23015428 PMC3541048

[B118] Zsurka G. Kunz W. S. (2015). Mitochondrial dysfunction and seizures: The neuronal energy crisis. *Lancet Neurol.* 14 956–966. 10.1016/S1474-4422(15)00148-9 26293567

